# Field assessment of potential sugar feeding stations for disseminating bacteria in a paratransgenic approach to control malaria

**DOI:** 10.1186/s12936-018-2516-x

**Published:** 2018-10-17

**Authors:** Etienne Bilgo, Amélie Vantaux, Antoine Sanon, Seni Ilboudo, Roch K. Dabiré, Marcelo Jacobs-Lorena, Abdoulaye Diabate

**Affiliations:** 1 Institut de Recherche en Sciences de la Santé/Centre Muraz, Bobo-Dioulasso, Burkina Faso; 2 Laboratoire d’Entomologie Fondamentale et Appliqué/UFR-SVT/Université Ouaga I, Pr. Joseph KI-Zerbo, Ouagadougou, Burkina Faso; 3 Institut Pasteur of Cambodia, Phnom Penh, Cambodia; 4 Department of Molecular Microbiology and Immunology, Malaria Research Institute, Johns Hopkins Bloomberg School of Public Health, Baltimore, MD 21205 USA

**Keywords:** Attractive sugar bait, Paratransgenesis, *Anopheles*, Open field, Malaria

## Abstract

**Background:**

Using bacteria to express and deliver anti-parasite molecules in mosquitoes is among the list of genetic tools to control malaria. The introduction and spread of transgenic bacteria through wild adult mosquitoes is one of the major challenges of this strategy. In prospect of future field experiments, an open field study with blank (without bacteria) attractive sugar bait (ASB) was performed under the assumption that transgenic bacteria would be spread to all sugar fed mosquitoes.

**Methods:**

Two types of ASB stations were developed, one with clay pots (CP) placed at mosquito resting sites and one with window entry traps (WET) placed inside inhabited houses. The ASB consisted in either glucose, honey or fruit cocktail solutions. In addition, mark-release-recapture (MRR) experiment of mosquitoes after feeding them with glucose was also conducted to check the proportion of the mosquito population that can be reached by the two ASB stations as well as its suitability to complement the ASB stations for disseminating bacteria.

**Results:**

Overall, 88% of the mosquitoes were collected in the WET_ASB. The CP_ASB stations were much less attractive with the highest average of 82 ± 11 mosquitoes/day in the CP near the wood piles. The proportions of sugar fed mosquitoes upon ASB were low in both type of ASB stations, ~ 2% and ~ 14% in WET and CP, respectively. Honey solution was the most attractive solution compared to the glucose and the fruit cocktail solutions. The recapture rate in the MRR experiment was low: ~ 4.1% over 7 days.

**Conclusion:**

The WET_ASB looks promising to disseminate transgenic bacteria to endophilic West Africa *Anopheles* mosquito. However, this feeding station may not be fully effective and could be combined with the CP_ASB to also target outdoor resting mosquitoes. Overall, efforts are needed to improve the mosquito-feeding rates upon ASB.

## Background

Malaria control currently relies on both the prompt diagnostic and effective treatments of malaria cases as well as vector control through insecticide-treated bed nets and indoor residual spraying [1]. Unfortunately, both parasites and vectors have developed resistance against many commonly used drugs and insecticides threatening the progresses made against malaria [2]. The development of new drugs, insecticides as well as a potential vaccine will require time and consequently highlights the need to also elaborate alternative control strategies [3]. However, these new approaches as totally novel tools are also a more risky strategy.

With the advent of modern molecular biology, genetic manipulations have been proposed as a new method to control the vector arthropods responsible for pathogen transmission to humans and animals [4]. While much work has focused on the direct genetic manipulation of vectors, implying the development of a transgenic strain for each vector species, an alternative strategy targeting symbiotic bacteria colonizing a large array of mosquito species would bring much more advantages by the use of a single transgenic strain in several vector species [4–6]. As originally formulated, paratransgenesis refers to engineering bacteria. However, the term has now been broadened to include other microbial agents, such as viruses and fungi [7]. Instead of killing the vector, this approach aims at blocking transmission through anti-pathogen molecules within mosquitoes. Past evidences suggest that this strategy works successfully in the laboratory [5, 8–10]. However, translation to the field requires several milestones to be reached. The first steps have been successfully completed with the development of a method for genetically engineering the vectors, the identification of effector molecules capable of killing the parasite and the identification of tissue-specific promoters to express the effector molecules at the appropriate time and place within the vector [11–13]. The next step now resides in driving transgenes into wild mosquito populations. How to introduce and spread transgenic bacteria and maintain them in wild mosquito population while also ensuring biosafety [12]? Mosquitoes naturally acquire most of the promising candidate bacteria for paratransgenesis, such as *Asaia* spp, *Serattia* spp and *Pantoa agglomerans.* Once a mosquito hosts a bacteria it can contaminate other individuals through horizontal transmission via shared feeding site (e.g. plant nectar) or mating, as well as contaminate its offspring through vertical transmission [14, 15]. However, in nature, while mosquito microbiota forms during the larval stages [16], individuals loose more than 95% of their total microbiota during pupation [14, 17]. Thus, young adult mosquito will obtain new bacteria during sugar feeding on plant nectar, such as e.g. *Phytotelmata*, *Plumbago auriculata* [14] or during resting at different sites [16]. Laboratory experiments have shown that transgenic bacteria can be spread through sugar meals [14, 18, 19] offering a potential tool for their introduction in the field. In addition, a previous laboratory investigation using sugar feeding as a means of re-introducing bacteria into adult mosquito midguts showed that mosquitoes did not discriminate between sterile sugar solutions and sugar solutions with bacteria [20]. However, no study has yet investigated how to introduce and disseminate bacteria through sugar meals in a field situation.

This open field study was carried out without any bacteria (blank sugar meal formulations only) as the prerequisite of assessing the efficacy and feasibility of this tool is necessary before developing a paratransgenic approach to control malaria. The overall aim was to develop different sugar meal feeding stations in order to target as much mosquitoes as possible while also representing different ages, status and genders to maximize the potential of bacteria dissemination. More specifically this study aimed at assessing the effectiveness of these feeding stations to attract wild mosquitoes and feed them. Therefore, attractive sugar bait (ASB) inside clay pots (CP) [21, 22] and window entry traps (WET) [23] were used. The two types of feeding stations (CP and WET) might not be informative enough to give the proportion of the total mosquito population in the village that can be reached by these methods of dissemination. Therefore, a mark release-recapture (MRR) trial was also performed in the same village before the onset of the rains. MRR coupled with glucose feeding was used to assess the efficacy of this technique to spread bacteria to wild mosquitoes.

## Methods

### Study area

**Fig. 1:**
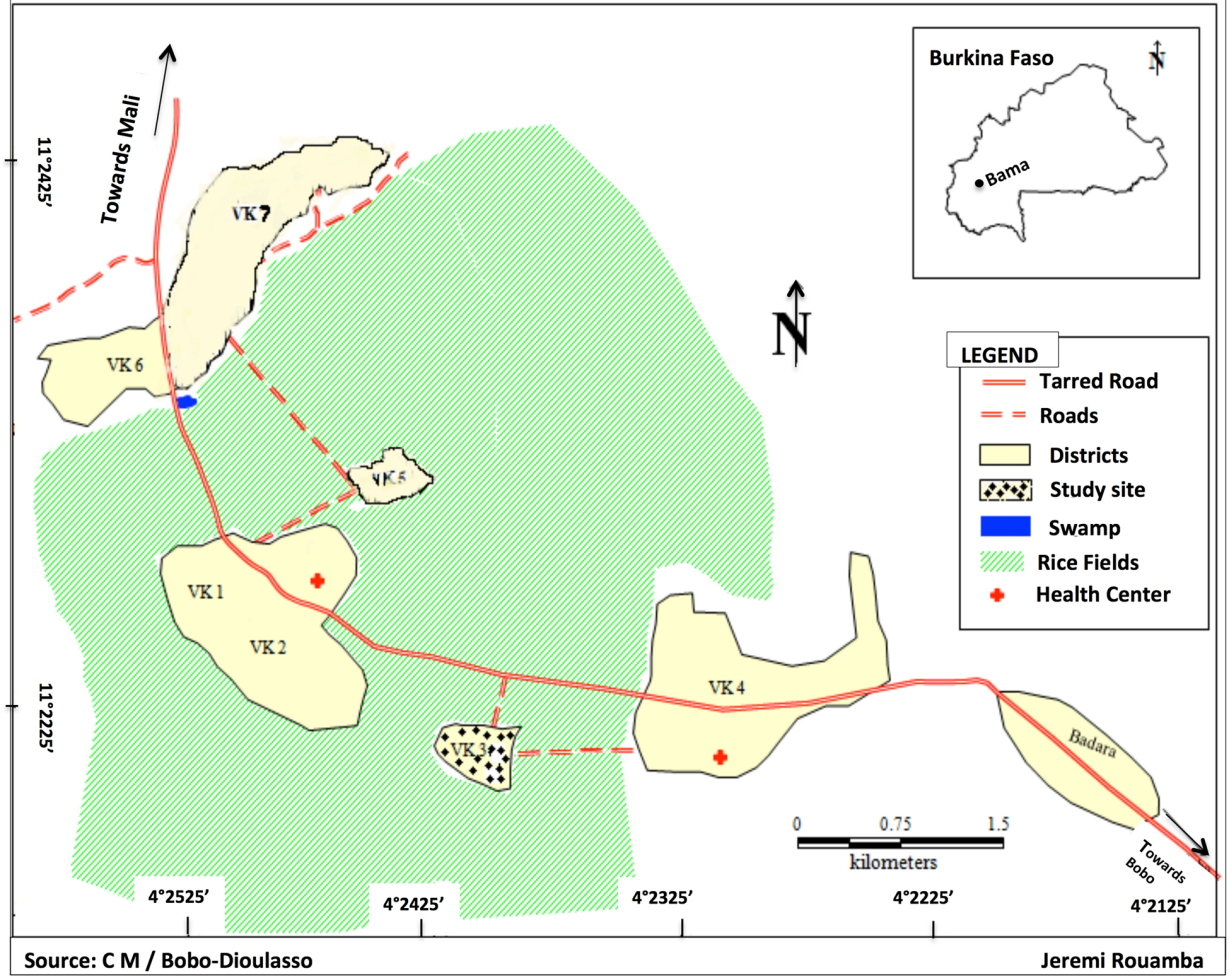
Study area

The study was carried out in Vallée du Kou in both the dry season in March 2013 for the MRR experiment and during the rainy season from July to September 2012 for the CP and WET experiments. The Kou Valley is a rice-growing area located about thirty kilometres North of Bobo Dioulasso (Southwestern Burkina Faso). It covers 1250 ha and is situated between 10° and 11° 55″ 25″ N and 4° 20 and 4° 35″ W. There are 7 villages with an estimated population of 18,000 inhabitants in 2010 [24]. Rainfall is about 1200 mm/year. This area is characterized by two seasons, the rainy season extends from May to October and the dry season from November to April. The Kou River is a permanent source of irrigation water for two crops of rice campaign per year. Rice growing provides highly productive mosquito larval breeding sites. Malaria transmission is first caused by *Anopheles gambiae* sensu lato *(s.l.)* and secondarily by *Anopheles funestus* in Bama [24]. The distribution of *Anopheles coluzzii* and *Anopheles gambiae* sensu stricto (*s.s.)* is subject to spatio-temporal dynamics with a predominance of *An. coluzzii* throughout the year [24]. This study took place in Ward 3, VK3 (Fig. 1).

### Preparation of attractive sugar meals

Three types of sugar meal solution were used for this study. Organic food dyes without any sugar and available in the local market in Burkina Faso were added to identify ASB fed mosquitoes versus unfed ones through visual inspection of distended dye-stained abdomens. It was previously confirmed in the laboratory that the colour of food dye did not have any attractive effects and that mosquitoes could be accurately differentiated based on the sugar meal they fed on (Bilgo, unpublished results). Cotton pads were soaked in each of the sugar solution. New solutions were prepared and cottons were replaced daily.

#### Glucose formulation

50 g of glucose was dissolved in 1 L of spring water. Red food dye was added and the solution was homogenized by shaking vigorously and manually for 5 min.

#### Honey formulation

50 g of natural honey was dissolved in 1 L of spring water. Yellow food dye was added and the solution was homogenized as above.

#### Cocktail of fruits formulation

A fruit cocktail of 300 mL of papaya juice (30%), 300 mL of watermelon juice (30%), and 250 mL of spring water (25%), 120 g brown sugar (12%) and 30 mL of local millet beer made with red sorghum (3%) was prepared. This cocktail was prepared based on previously observed mosquitoes preferences as well as enhanced attractiveness thanks to the C02 from the local beer [25]. No food dye was used in this solution as the cocktail already had a bright red-yellow colour.

### Clay pots attractive sugar bait stations

**Fig. 2:**
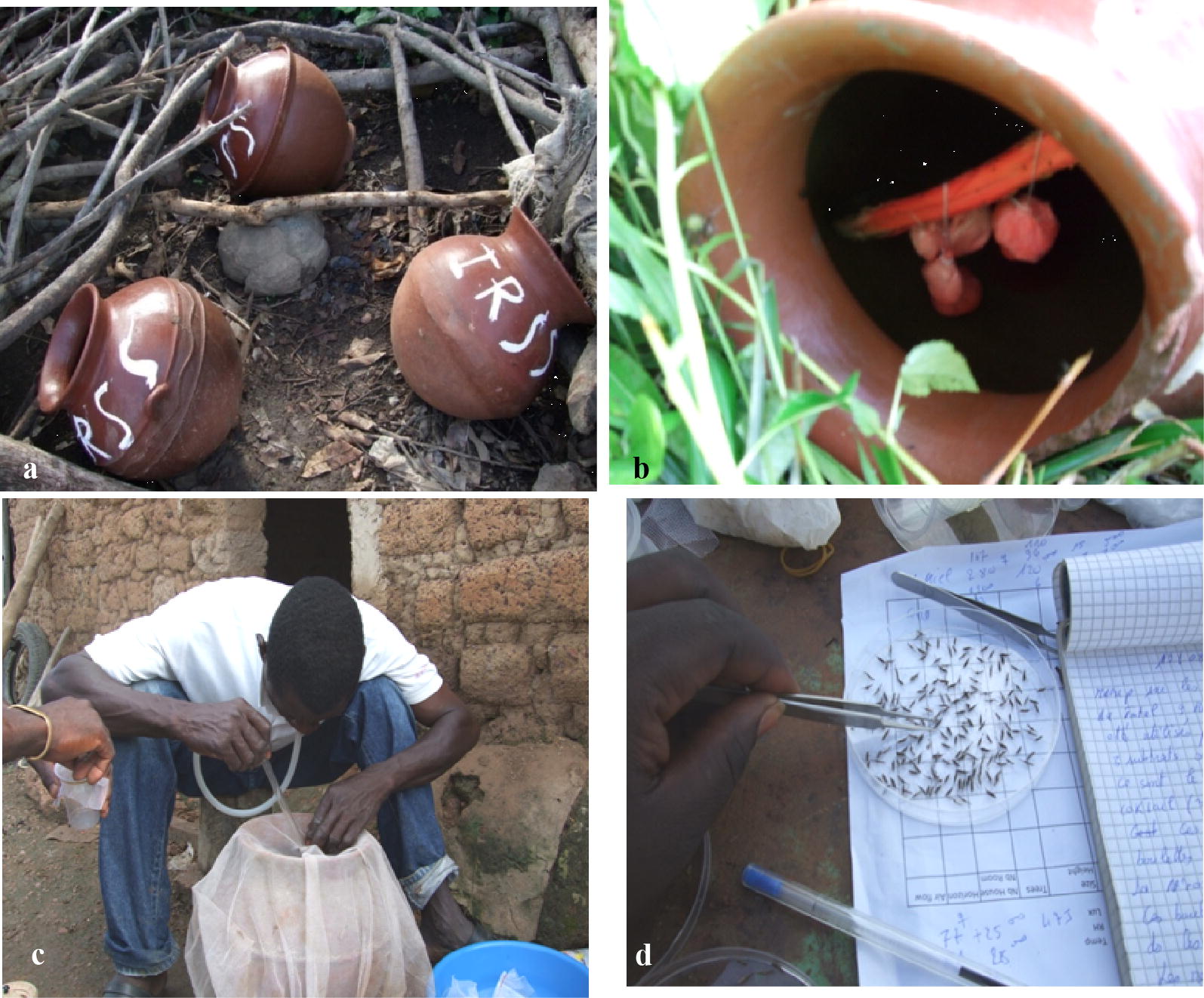
Attractive sugar feeding station with clay pots (CP_ASB). **a** Three clay pots containing cotton pads soaked with the sugar solutions and deployed near wood piles. **b** Clay pot containing cotton pads soaked in the glucose. **c** Manipulation of a clay pot to retrieve mosquitoes resting in it. **d** Morphological determination of mosquitoes at the study site

Clay pots are conventionally used for storing drinking water in the homes in the study area, and were of local design and manufacture. Each pot was of ~ 20 L capacity, with an opening of 20 cm width, a round bottom, and a maximum width of 45 cm (Fig. 2a, b). Four different types of resting sites were targeted: inhabited houses, unoccupied houses, outdoor resting site such as wood piles within 5 m of each sampled house and resting sites next to larval sites (Fig. 2a). For each type of resting site, three CP were set-up (one for each sugar solution) and each CP contained 4 pairs of soaked cotton pads. In addition, for each type of resting site the clay pots were set-up in 3 different areas of the village, each area being located around 300 m from each other. Therefore, a total of 36 CP were dispatched throughout the village. The experiment was conducted for 4 days/month from July to September. Mosquitoes were sampled daily from the clay pots. A cloth mesh was placed over the clay pot to prevent mosquito from escaping and a hand held mouth aspirator was introduced through a 2 cm hole to collect resting mosquitoes (Fig. 2c). Any spider webs and organisms were brushed out. Collections were carried out between 6 a.m. and 9 a.m. The feeding status of each individual was determined through visual examination of the abdomen. After morphological identification and counting (Fig. 2d), the samples were stored in 70% ethanol for further species identification for sibling species (*An. gambiae s.s.*, *An. coluzzii* or *Anopheles arabiensis*) using routine RFLP-PCR as in [24].

### Window entry trap attractive sugar bait station

**Fig. 3:**
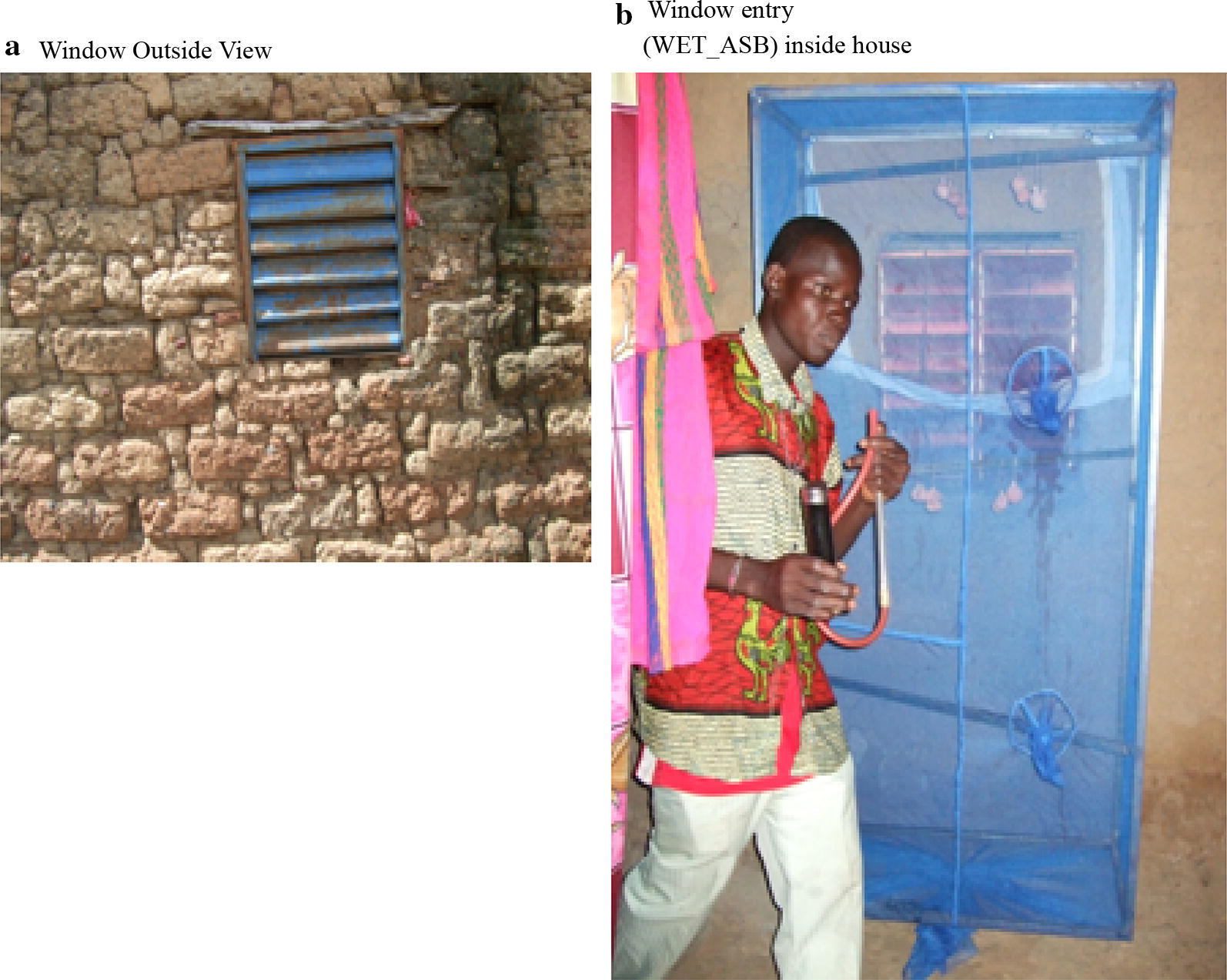
Attractive sugar feeding station with window entry trap (WET ASB), **a** Outside view (Window); **b** WET equipped with glucose soaked cotton pads installed inside a house

The WET was made with a metal frame and fitted from the bottom to the top with regular mosquito nets. Each window entry trap measured about 1.70 m × 0.85 m × 0.5 m. However, the height was variable depending on the type of construction. The window entry traps were fixed indoors ([26]; Fig. 3). A total of 9 WET were dispatched within the village (1 WET/house). The village was subdivided in 3 areas 300 m away from each other. In each area, 3 inhabited houses 5 m away from each other were selected. The trial was conducted over 4 days monthly from July to September. Four pairs of cotton pads soaked in each of the 3 solutions were hanged in each trap each evening between 6 p.m. and 7 p.m. The windows were opened in the evening (6 p.m.–7 p.m.) until early morning (5.30 a.m.–6 a.m.) and closed during the day. Mosquito collections were carried out in the morning after closing the window using hand-held manual aspirators. Mosquito feeding status and morphological identification were carried out as above. A randomly selected subsample was then stored in 70% ethanol for future molecular analyses as above.

### Mark release-recapture

**Fig. 4:**
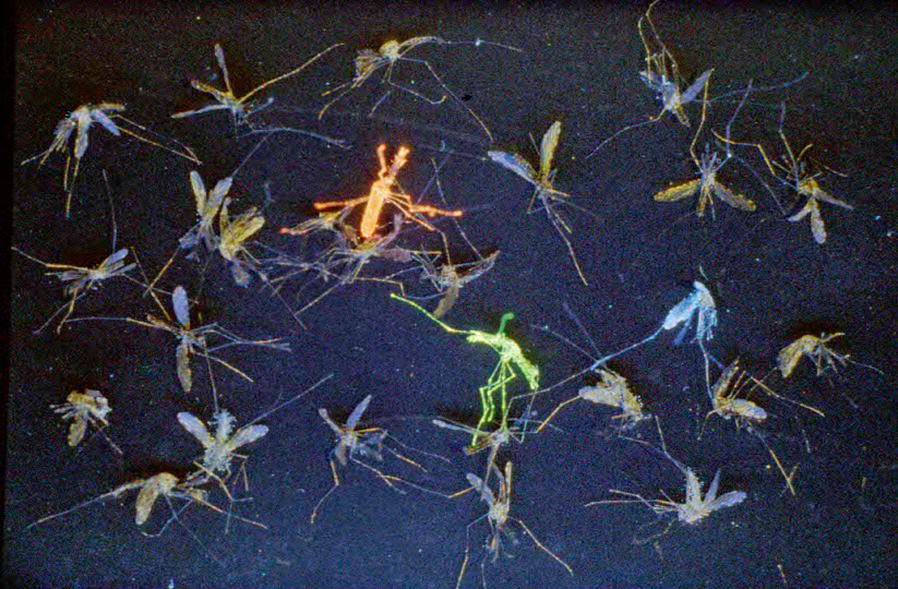
Fluorescent dusted mosquitoes in the mark-release-recapture trial

Mark-release-recapture was carried out during the dry season in March for 7 consecutive days. Female mosquitoes were collected from indoor of 20 inhabited houses, dusted with different fluorescent powder for each day of release (Green, yellow, blue, orange, green, yellow, blue for the 7 days, respectively) and fed on a 5% glucose solution. Marked-sugar-fed mosquitoes were released ~ 10–12 h after capture within 5 m from their point of capture in the village. The rationale behind this study is that if the daily rate of recruitment of new emerging adults is lower than our sampling effort, we will expect an increasing proportion of dusted adults in the total collection day after day. Recaptures of mosquitoes were performed indoor houses every morning between 6 and 9 a.m. The numbers of dusted and non-dusted mosquitoes were scored (Fig. 4).

### Data analysis

Only *An. gambiae s.l.* mosquitoes were considered for analyses. The total number of mosquitoes was compared using a Generalized Linear Mixed Model (GLMM) with a Poisson error structure. In this model, the type of resting site (WET, CP inhabited houses, CP unoccupied houses, CP wood pile, CP larval breeding sites), sex (male or female), sugar-fed status (fed or not on ASB) and their interaction were coded as fixed factor. The proportion of fed mosquitoes was compared using a GLMM with a binomial error structure. In this model, the type of resting site, sex and their interaction were coded as fixed factor.

A data subset including ASB fed individuals only was used to compare the number of fed mosquitoes on each type of ASB solution using GLMM with a Poisson error structure. The type of resting site, sex, type of ASB solution (glucose vs. honey vs. cocktail) and their interaction were coded as fixed factor.

A data subset that included females only was used to compare the effect of the gonotrophic status using a GLMM with a Poisson error structure. In this model, the type of resting site, their gonotrophic status (ASB fed *vs*. blood fed *vs.* gravid *vs.* unfed), and their interaction were coded as fixed factor.

In all these models, replicate and individual were coded as random factors to account for repeated measures and for over dispersion, respectively. For model selection, we used the stepwise removal of terms, followed by likelihood ratio tests. Term removals that significantly reduced explanatory power (P < 0.05) were retained in the minimal adequate model [27]. All analyses were performed in R v.3.0.3.

## Results

### Performances of clay pot and window entry trap attractive sugar bait

A total of 83,189 mosquitoes were collected over the 12 collection days. Overall, 88% of the mosquitoes were collected in the WET. When considering the CP only, 77% of the mosquitoes were caught in unoccupied houses and near wood piles. On average, more females were collected per day (616 ± 151) than males (78 ± 13; X_1_^2^ = 587, P < 0.0001). Similarly, the average number per day of mosquitoes not feeding on ASB was higher (669 ± 149) than the ones fed on ASB (24 ± 3; X_1_^2^ = 2694, P < 0.0001). There was a significant effect of the type of resting site on the average number per day of collected mosquitoes (X_4_^2^ = 2876, P < 0.0001). On average 21 ± 3, 27 ± 4, 77 ± 10, 82 ± 11 and 1526 ± 339 mosquitoes/day were collected in the CP placed in the inhabited house, near the larval breeding sites, in the unoccupied houses, near the wood pile and in the WET, respectively. The average number of mosquitoes collected per day was significantly affected by the interaction between the collection site and the sugar feeding status (X_4_^2^ = 402, P < 0.0001, Table 1).

**Table 1:** Average number of ASB fed and unfed mosquitoes per day collected in the different types of ASB stations

ASB_feeding status	Types ASB stations	Average number of mosquitoes (± se)
ASB_fed	WET_inhabited house	63.08 ± 13.34
	CP_larval breeding site	7.96 ± 0.89
	CP_wood pile	22.08 ± 1.84
	CP_unoccupied house	21.75 ± 2.015
	CP_inhabited house	6.042 ± 0.78
Unfed	WET_inhabited house	2988.96 ± 533.6
	CP_larval breeding site	45.54 ± 5.2
	CP_wood pile	142.42 ± 11.93
	CP_unoccupied house	132.54 ± 11.45
	CP_inhabited house	35.83 ± 3.66

In particular, significantly more (~ 47-fold) unfed than fed mosquitoes were collected in the WET compared to the four types of CP (~ sixfold; Chi square post hoc tests P < 0.0001). The average number of mosquitoes collected per day was significantly affected by the interaction between the resting site and the mosquito sex (X_4_^2^ = 333, P < 0.0001; Table 2).

**Table 2:** Average total number and number of male and female mosquitoes (*Anopheles gambiae s.l.*) collected in the different feeding stations

Type of feeding stations	Location	Sex of mosquitoes	Average number of mosquitoes/day (mean ± se)
Window entry trap (W.E.T.)	Inhabited house	Females	2804.08 ± 571.07
		Males	247.96 ± 53.45
Clay pot (C.P.)	Larval breeding site	Females	35.88 ± 6.62
		Males	17.62 ± 2.73
	Wood pile	Females	103.67 ± 18.46
		Males	60.83 ± 8.94
	Unoccupied house	Females	105 ± 16.6
		Males	49.29 ± 7.67
	Inhabited house	Females	29.17 ± 4.75
		Males	12.71 ± 2.19

In particular, eleven times more females than males were collected in the WET, whereas only two times more females than males were collected in the CP inhabited and unoccupied houses, and similar number of males and females were collected in the CP near the larval breeding sites and the woodpiles (Chi square post hoc tests: P < 0.01). Forty-six times more females were unfed than fed, whereas only 22 times more males were unfed than fed (mosquito sex by sugar-fed status interaction; X_1_^2^ = 12, P = 0.0004). Finally, the average number of mosquitoes collected per day was significantly affected by the interaction between the resting site, the sugar feeding status and the mosquito sex (X_4_^2^ = 12, P = 0.02). However, post hoc comparisons were not significant.

Overall, 3.4 ± 0.7% (2902/83,189) of the collected mosquitoes fed on the ASB. Males feeding rate on ASB was significantly higher than the females feeding rate (7.6 ± 0.2% vs. 2.97 ± 0.1% respectively; X_1_^2^ = 17.35, P < 0.0001). The feeding rate was significantly affected by the type of resting sites (X_4_^2^ = 609, P < 0.0001) with the feeding rate in the WET (~ 2%) being significantly lower than the feeding rates in all other CP feeding stations (~ 14%; Table 3).

**Table 3:** Mosquito feeding rates upon ASB by resting sites and type of ASB

Type of ASB and location	Proportion of ASB fed (% ± 95% CI)
WET_inhabited house	2.07 ± 0.097
CP_larval breeding site	14.88 ± 0.24
CP_wood pile	13.42 ± 0.23
CP_unoccupied house	14.1 ± 0.24
CP_inhabited house	14.43 ± 0.24

**Fig. 5:**
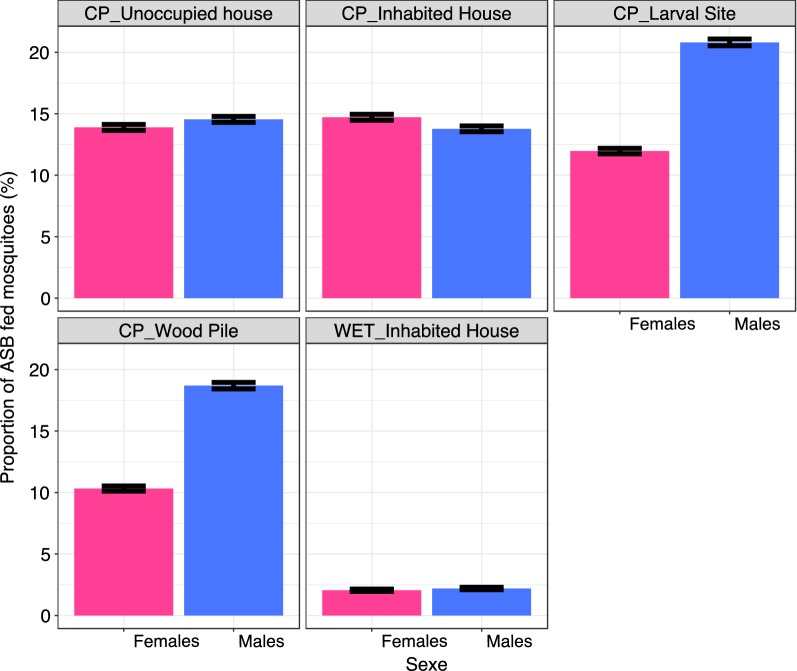
Proportions of ASB fed mosquitoes by sexes in the different Attractive Sugar Feeding Stations

The feeding rate was significantly affected by the interaction between sex and type of resting site (X_4_^2^ = 17.4, P = 0.002). In particular, there was a similar proportion of ASB fed females and males in the WET, CP unoccupied houses and inhabited houses, whereas the proportion of ASB fed males was higher than the proportion of ASB fed females in the CP near larval breeding sites and CP near wood piles (Fig. 5).

Among the 2902 ASB fed individuals, 31.8 ± 1.7% had fed on the cocktail solution, 40. 1 ± 1.8% fed on the honey solution and 28 ± 1.6% fed on the glucose solution. The type of ASB solution significantly affected the mean number of ASB fed individuals per day (X_2_^2^ = 30, P < 0.0001) with more mosquitoes fed on the honey (9.7 ± 1.3) compared to the cocktail (7.7 ± 1) and the glucose solutions (6.8 ± 1). On average, more ASB-fed females than males were collected per day (12.2 ± 1.2 *vs*. 3.9 ± 0.26, respectively, X_1_^2^ = 206, P < 0.0001). The resting sites significantly affected the mean number of ASB fed mosquitoes (X_1_^2^ = 623, P < 0.0001). On average, more ASB fed individuals were collected in the WET (21 ± 2.6) than in the CP wood pile (7.36 ± 0.5), in the CP unoccupied house (7.25 ± 0.45), in the CP larval breeding site (2.65 ± 0.2), and in the CP inhabited house (2 ± 0.2). All post-comparisons were significant except between the CP unoccupied and CP inhabited houses and between the CP larval breeding sites and CP wood pile. There was a significant interaction between the resting sites and mosquito sex (X_4_^2^ = 233, P < 0.0001). All post hoc comparisons were significant except between ASB-fed males and females in the CP unoccupied and the CP inhabited houses, and between the CP larval breeding sites and the CP wood pile. There was no significant effect of the interaction between resting site and type of ASB solution (X_8_^2^ = 8.1, P = 0.4), between type of ASB solution and mosquito sex (X_2_^2^ = 0.59, P = 0.75), nor of the three-way interaction between collection site, mosquito sex and type of ASB solution (X_8_^2^ = 4.7, P = 0.78).

**Fig. 6:**
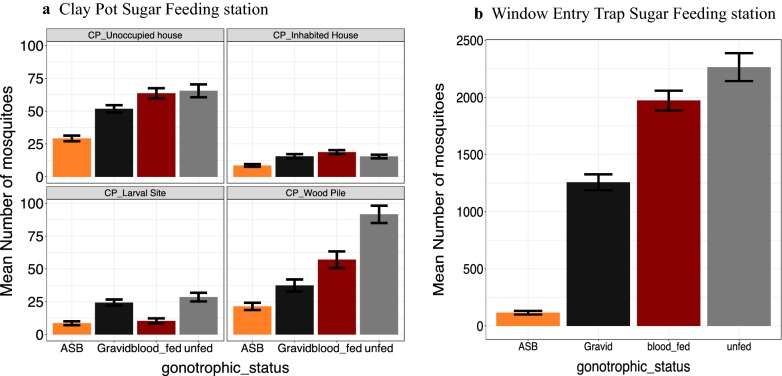
Average number of collected females per day by gonotrophic status in the different attractive sugar feeding stations

When considering the subset of females only (n = 73,867), the average number of collected females was significantly affected by the gonotrophic status (X_3_^2^ = 968, P < 0.0001). There was on average significantly more unfed (493 ± 118) than blood fed (424 ± 102), gravid (277 ± 65) and ASB-fed (37 ± 6) females collected per day (All Chi square post hoc tests were significant except between blood fed and gravid females). The average number of collected females was significantly affected by the resting sites (X_4_^2^ = 7115, P < 0.0001). On average 1402 ± 127 females were collected in the WET, 52 ± 5 in the CP woodpile, 52 ± 3 in the CP unoccupied houses and 18 ± 2 in the CP larval breeding sites and 15 ± 1 in the CP of inhabited houses (All Chi square post hoc tests were significant except between the CP woodpile and the CP unoccupied houses, and between the CP inhabited houses and CP woodpile). The average number of collected females was significantly affected by the interaction between resting sites and gonotrophic status (X_12_^2^ = 460, P < 0.0001; Fig. 6).

### Mark-release-recapture

**Fig. 7:**
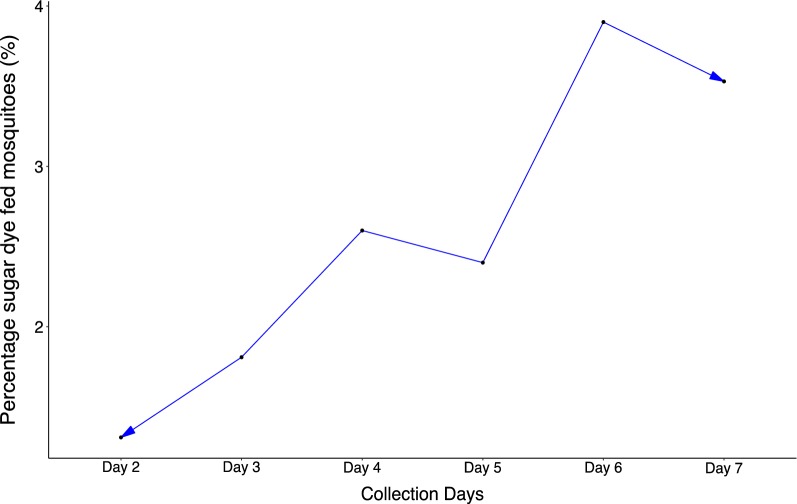
Proportion of sugar dye fed mosquitoes over the whole population in the MRR trial

In total 14,898 specimens were collected and of them, 11,525 were dusted, fed and released over 7 days (Table 4). A cumulative effect in our sampling effort could be observed over the 7 days of collection, though this cumulative effect was low and ranged from 1.3 to 3.90%, suggesting that over 7 days of release-recapture, nearly 4% of the mosquito population was reached (Fig. 7).

**Table 4:** Total mosquitoes collected in houses in VK3, fed and released in mark-release-recapture experiments

	Total mosquitoes collected	Total mosquitoes fed and released
Day 2	1750	1503
Day 3	2202	1897
Day 4	1987	1851
Day 5	1727	1625
Day 6	2412	2303
Day 7	2611	2346

## Discussion

In prospect to develop tools for introducing and disseminating genetically modified bacteria into wild mosquitoes when the paratransgenic approach will come on age, we assessed for the first time the use of ASB feeding station in association with two trapping tools, the clay pots and the window entry traps, in an open field study [23, 25–28].

For the ASB approach using clay pots, the unoccupied houses and the wood piles were found to be the best-resting sites among the four tested in this study. Since these two sites resulted in the highest yield of mosquitoes, they should be targeted to disseminate bacteria for paratransgenesis. The fact that a low number of mosquitoes were collected in inhabited houses when using the CP could be due to the presence of more attractive resting sites to the individuals in these houses (e.g. ceiling, other containers, etc.). *Anopheles gambiae* is known to be highly endophagous and endophilic [23]. However, another explanation could be that females fed indoors and then exited the houses following behavioural changes due to LLINs use [29]. Indeed, the mass distribution of insecticide-treated nets has selected a fraction of endophagous and exophilic mosquitoes in East Africa [30, 31]. However, it does not seem to be the case yet in the Vallée du Kou [32]. Several studies on malaria transmission have shown that more than ¾ of mosquitoes are collected indoors in this study area [23, 33, 34]. Similarly to inhabited houses, the CP next to larval breeding sites might have yield a low number of mosquitoes as there are probably more alternatives than the CP feeding station to rest and feed. A previous study in Mali, demonstrated that a large portion of emerging mosquitoes rested on the grasses close-by the breeding sites and fed directly on nearby plants and wild grasses [21]. Accordingly, the clay pots that have been dispatched would be less competitive in this specific location and their design should be improved to attract more mosquitoes.

The low feeding rate of mosquitoes on all proposed ASB suggests that these sugar meals were either not attractive enough for mosquitoes or that the formulation, the cotton pads are not suitable to let mosquito feed upon them. However recent lab studies used these cotton pads soaked with sugar solution and bacteria to infect laboratories *Anopheles* strains [18, 35]. Could wild mosquitoes prefer to feed on natural sugar nectars rather than on our artificial sugar meals especially as they were free to choose? Overall, mosquitoes preferred to feed upon the honey compared to the glucose and the cocktail solutions. On the contrary, previous studies showed that mosquitoes prefer to feed on fruit such guava and honey melon which were rotten [32, 36].

Clay pots have several advantages. They are cool and dark, offering a protection from sun radiations and dehydration to mosquitoes. The clay pots were useful and practical for sampling both sexes and all gonotrophic status of *An. gambiae s.l.* In addition, clay pots are low cost, durable and self-operational devises. Therefore, they would be well suited for delivering contaminated sugar meal with bacteria to outdoors male and female mosquitoes. However, clay pots also bare some disadvantages. First, besides mosquitoes they also provide resting places for other animals, such as lizards, spiders, and scorpions, some of which are potential mosquito predators. The regular inside-brushing of the pots could solve this issue. A second limitation of the clay pot feeding station is the impossibility to estimate the fraction of mosquitoes touched daily in the village. Consequently, this approach will hamper the projection over time of the dissemination of bacteria into wild mosquitoes.

In parallel to the CP approach, we tested the efficacy of WET to disseminate transgenic bacteria through ASB station. One important advantage of this approach is that it targets the most important mosquitoes in malaria transmission: the endophilic and anthropo-endophagic ones. However, more than with clay pots, the sugar meal-feeding rate was very low limiting the spread of transgenic bacteria. As for clay pots, the different ASB solutions might not have been attractive enough. In addition, more females than males were collected in this trap, which is likely explained by the fact that females are more likely to enter inhabited houses to have a blood meal or rest rather than to obtain a sugar meal [37]. Interestingly it should be noted that a video-surveillance study was conducted with a camera to check the behaviour of mosquitoes in the WET just after fixing the traps and ASB from 6 p.m. to 6 a.m. The video-surveillance has demonstrated a higher sugar meal-feeding rate than the one observed in this experiment. This rate was ~ 22% conversely to ~ 2% in the control cage. Therefore, a large fraction of mosquitoes got their sugar meal in the WET and then left. Further studies using this protocol of surveillance are needed to elucidate this issue of feeding rate in the WET. The WET as a feeding station to introduce and disseminate bacteria has as advantage that it is simple and easy to use. It is cheaper to construct and does not require heavy logistics and personnel. It is a tool that works itself’. In addition, this approach gives a good estimate of the fraction of mosquitoes targeted out of the total mosquito in the village. Although a couple of alternative outdoor resting sites for mosquitoes exist, the WET will allow to contaminate and spread the bacteria in short term to epidemiologically important mosquitoes. However, efforts are needed to improve the attractiveness of sugar meals to mosquitoes in the WET.

With the third approach, the MRR, a cumulative effect was expected over time. This means that the daily collection efforts in the village should be higher than the emergence of new females. Three conditions are necessary to have a better recapture rate. Firstly, mosquito density must be very low. Secondly, the number of active breeding sites in the village has to be limited. Thus, the number of daily emerging adults will be lower than the collection efforts. Thirdly, the study area has to be isolated from the other sites and to be relatively small with a limited number of houses in order to allow their daily screening for the mosquito collections. Such method could be applicable in small semi-arid area and/or during the dry season. In this study, unfortunately the mosquito density was already high in March despite being in the dry season. Several larval breeding sites already functioned due to some scattered rain falls in the village. Consequently, we were unable to cover all the houses in the village every day. Moreover, no collection were conducted in the outdoor resting sites which could have increased our recapture rate. The transmission of most of promising bacteria, such as *Asaia* spp., *Serratia* spp., *Pantoa Agglomerans*, etc. for the paratransgenic approach is horizontal and vertical within mosquitoes [14, 15, 35]. In theory, just a fraction of the mosquito population needs to be contaminated with those bacteria in order to be spread out through the rest of the mosquito population. The advantage of this approach is that it gives a good idea of the proportion of mosquitoes that we have fed and released. However, the approach is not self-functional, as it requires daily big mosquito collection efforts.

## Conclusion

The present study shows that the WET sugar feeding stations are the most promising tool to introduce and spread bacteria through the mosquito population. However, CP sugar feeding station could also be suitable at some point or integrated to the WET feeding stations approach. The implementation of the two tools tested (WET and CP) is relatively low technology and independent of mosquito or parasite species; however, one of the future challenges will be to increase the sugar feeding rate. In this study, no bacteria were used as it was assumed that all mosquitoes fed upon the sugar meal would be contaminated with the bacteria. Thus, a second step will be to evaluate the actual dissemination of transgenic bacteria in a semi field setting using the tools and methods developed here. Overall, these results will provide critical information on the effectiveness of various approaches to introduce bacteria into mosquitoes in the field and the extent of spread through mosquito populations, which are essential steps in the development of this technic for malaria control.

## Abbreviations

ASB: attractive sugar bait
CP: clay pot
WET: window entry trap
VK3: Vallée du Kou (Kou Valley), village number 3
MRR: mark release-recapture
GLMM: Generalized Linear Mixed Model
